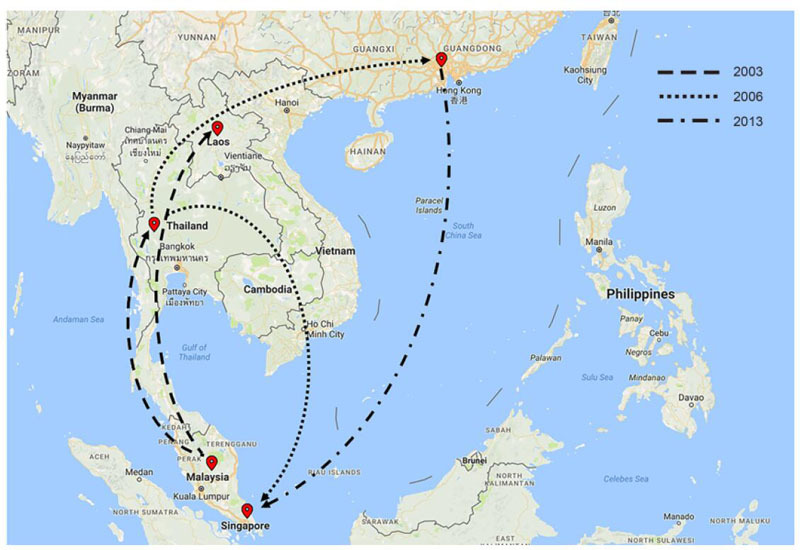# Molecular epidemiology demonstrates that imported and local strains circulated during the 2014 dengue outbreak in Guangzhou, China

**DOI:** 10.1007/s12250-016-3872-8

**Published:** 2017-01-23

**Authors:** Geng Li, Pan Pan, Qiuyan He, Xiujuan Kong, Kailang Wu, Wei Zhang, Yuntao Liu, Huiting Huang, Jianbo Liu, Zhongde Zhang, De Wu, Xiaoping Lai, Xiaohong Liu, Jianguo Wu

**Affiliations:** 1 School of Chinese Meterla Medica, Guangzhou University of Chinese Medicine, Guangzhou, 510006 China; 2 State Key Laboratory of Virology, College of Life Sciences, Wuhan University, Wuhan, 430072 China; 3 The First Affiliated Hospital of Guangzhou University of Chinese Medicine, Guangzhou, 510120 China; 4 Guangdong Province Traditional Chinese Medical Hospital, Guangzhou, 510120 China; 5 Guangdong Provincial Center for Disease Control and Prevention, Guangzhou, 511430 China

**Keywords:** dengue virus (DENV), phylogenetic analysis, envelope (*E*) gene, enzootic transmission cycle

## Abstract

The dengue virus (DENV) is a vital global public health issue. The 2014 dengue epidemic in Guangzhou, China, caused approximately 40,000 cases of infection and five deaths. We carried out a comprehensive investigation aimed at identifying the transmission sources in this dengue epidemic. To analyze the phylogenetics of the 2014 dengue strains, the envelope (*E*) gene sequences from 17 viral strains isolated from 168 dengue patient serum samples were sequenced and a phylogenetic tree was reconstructed. All 17 strains were serotype I strains, including 8 genotype I and 9 genotype V strains. Additionally, 6 genotype I strains that were probably introduced to China from Thailand before 2009 were widely transmitted in the 2013 and 2014 epidemics, and they continued to circulate until 2015, with one affinis strain being found in Singapore. The other 2 genotype I strains were introduced from the Malaya Peninsula in 2014. The transmission source of the 9 genotype V strains was from Malaysia in 2014. DENVs of different serotypes and genotypes co-circulated in the 2014 dengue outbreak in Guangzhou. Moreover, not only had DENV been imported to Guangzhou, but it had also been gradually exported, as the viruses exhibited an enzootic transmission cycle in Guangzhou.

## INTRODUCTION

Dengue virus (DENV) is a major health challenge in tropical and subtropical countries (Zellweger et al., 2010), where an enzootic cycle occurs between the order *Primates* and *Aedes* mosquitoes. In humans, the clinical spectrum of DENV infection ranges from asymptomatic infection to dengue fever (DF), dengue hemorrhagic fever (DHF), dengue shock syndrome (DSS) and death (Cruz et al., 2013). The vast distribution of mosquito vectors, which is fueled by frequent human migration, and inadequate listed vaccines and effective drugs, have resulted in a dramatic increase in DENV infections, which have undergone 30-fold growth over the last 50 years. The World Health Organization has estimated that more than 50–100 million DENV infections occur in over 100 countries annually, putting almost 2.5 billion people at risk (Costa et al., 2012).

DENV is a single-stranded, positive-sense RNA virus that belongs to the family *Flaviviridae*. Its genome is approximately 11, 000 nucleotides, and encodes three structural proteins, including the C (core), M (membrane) and E (envelope) proteins; seven nonstructural proteins, including NS1, NS2a, NS2b, NS3, NS4a, NS4b and NS5 (Drumond et al., 2012); and short non-coding regions at both the 5′- and 3′-ends (WHO, 2009; Rodenhuis-Zybert et al., 2010). DENV can be grouped into five antigenically distinct serotypes (DENV-1, -2, -3, -4 and -5) (Wang et al., 2015) and each of these serotypes can be sub-classified into several genotypes based on their E gene sequences. Various genotypes have been epidemic in different geographic regions (Holmes and Burch, 2000). DENV-1, -2, -3 and -4 have been found and been prevalent in China.

The DENV-1 serotype can be classified into five genotypes, I, II, III, IV and V (Villabona-Arenas and Zanotto, 2013). Genotype I strains have been collected throughout Southeast Asia, China and the Middle East, while genotype II has been collected only in Thailand during the 1950s and 1960s. Genotype III was collected in Malaysia in 1972, while genotype IV is from Pacific Rim countries (Japan, Korea, China, Myanmar, Malaysia and Indonesia), The Western Pacific islands (for example, French Polynesia, Nauru, The Philippines and Hawaii) and Australia. Genotype V has been collected in the Americas, as well as from West Africa and Asia.

DENV-2 has six genotypes, consisting of Asian I, Asian II, Southeast Asian/American, Cosmopolitan, American and sylvatic (Sang et al., 2015; Cruz et al., 2013; Mir et al., 2014). Additionally, DENV-3 can be arranged into five genotypes, including I–V. DENV-4 is classified into four genotypes, consisting of I–III and the sylvatic genotype. Moreover, a new serotype of DENV-5 was isolated in Malaysia in 2013.

In ancient China, dengue-like illness was described as "water poison, " as it was associated with aquatic insects dating back to the Jin, Tang and early Song Dynasties (Common Era 265–420, 610 and 992 AD, respectively). Chinese modern history began in 1840, and it was in 1873, the 1920s and the 1940s that DF was clearly reported in the coastal provinces of China; three decades later, the first DF outbreak was identified in 1978. The outbreak occurred in Guangdong province, where serotype 4 (DENV-4) reemerged, causing 22, 122 infections and 14 deaths. Since then, sporadic outbreaks have occurred in the Guangxi, Fujian, Hainan and Yunnan provinces, where DENV-1, -2, -3 and -4 have been detected.

Guangzhou, with a population of 13 million, is the provincial capital of Guangdong province, and the transportation, industrial, financial and trade center of South China (Sang et al., 2015). As Guangzhou is an important entry/exit port in South China, there is a high population migration between it and Southeast Asia, where DENV originated. Guangzhou has a subtropical monsoon climate with suitable habitats for *Aedes albopictus* (DENV transmission vector). Taken together, by 2009, nearly 20, 000 DF cases had been reported in Guangzhou, accounting for 67% of the DF cases in Guangdong province and 56% of those in China (Luo et al., 2013).

Before 2013, a molecular epidemiology study of DENV in Guangzhou showed that dengue in China still remained an imported disease. In this study, we propose that a localization of DENV has gradually emerged. We identified envelope (*E*) gene sequences from 17 dengue isolates collected in Guangzhou during the 2014 outbreak. We conducted an extensive molecular epidemiological analysis of these sequences in combination with sequences published in GenBank that was aimed at determining where the DENV strain that caused the 2014 Guangzhou pandemic originated (Wu et al., 2011).

## MATERIALS AND METHODS

### Sample collection

Patient samples for this study came from The First Affiliated Hospital of Guangzhou University of Chinese Medicine and Guangdong Province Traditional Chinese Medical Hospital. All patients aged between 18- and 75-years-old with fever > 37.5 °C for less than 72 h were invited to participate in the study. Anti-dengue IgM and IgG enzyme-linked immunosorbent assay kits were employed to confirm dengue infection (Radke et al., 2012). We obtained 168 serum samples during the dengue outbreak in Guangzhou in 2014.

### Virus isolation, RNA extraction and serotyping

DENV isolation was conducted from the patients' positive blood samples. To increase virus titer, we first used an *Ae. albopictus* gut cell line (C6/36) for virus propagation. The C6/36 cell line was cultured in MEM medium (Gibco, Carlsbad, CA, USA) supplemented with 10% fetal bovine serum (FBS; Gibco, Carlsbad, CA, USA) for cell maintenance. Patient serum was diluted 10-fold in fresh MEM medium. After the addition of 200 μL serum to the C6/36 cells ($1.5\times10^5$ cells), the cells were incubated at 37 °C for 90 min. Then, the medium was replaced with fresh MEM medium with 2% FBS; the cells were maintained at 28 °C with 5% $CO_2$ for 5 to 7 days (until significant cytopathic effects (CPE), due to infection, were present). Simultaneously, 400 μL supernatants were collected for RNA extraction after centrifugation at 4000 rpm. These samples were then stored at –80 °C until needed.

TRIzol reagent (Invitrogen, Carlsbad, CA, USA) was used to extract RNA according to the manufacturer's in-

Table 1. Primers used for amplifying and sequencing the complete DENV-1 envelope gene

| Reaction | Primer name | Sequence (from 5′ to 3′) | Position |
|---|---|---|---|
| Amplification | DENV E1 Forward | TGCCATAGGAACATCCATCAC | 863–883 |
| Amplification | DENV E2 Reverse | TCCCAATGGCTGCTGATAGTC | 2495–2462 |
| Sequencing | DENV E1 Forward | TGCCATAGGAACATCCATCAC | 863–883 |
| Sequencing | DENV E2 Reverse | TCCCAATGGCTGCTGATAGTC | 2495–2462 |
| Sequencing | DENV E3 | CGGAAAAGGTAGCCTAATAACG | 1259–1280 |

structions; then, the RNA was reverse-transcribed into cDNA, at 42 °C for 60 min followed by 72 °C for 10 min, with reserve transcriptase. Finally, DENV serotyping was carried out by multiplex RT-PCR (Yong et al., 2007).

## Sequencing

After cDNA reverse transcription to make the templates, RT-PCR was performed to amplify the envelope (*E*) gene (1485 bp). The PCR reaction included 0.3 μmol/L each primer, 1 μL *Thermococcus kodakaraensis* (KOD)-Plus-Neo polymerase (Toyobo Co., Ltd, Osaka, Japan), 5 μL 10× PCR buffer, 1.5 mmol/L $MgCl_2$, 0.2 mmol/L dNTPs and 2 μL cDNA. The PCR amplification consisted of 40 cycles of 10 s denaturation at 98 °C for 30 s, annealing at 58 °C for 50 s and extension at 68 °C for 50 s, and a final extension at 68 °C for 10 min. The amplicons were purified with Centri-Sep columns (Invitrogen, Carlsbad, CA, USA). Double-stranded sequencing of the E gene was performed on an ABI PRISM 3700 Genetic Analyzer (Applied Biosystems Inc, Foster City, CA, USA) using an ABI PRISM BigDye Terminator Cycle Sequencing Ready Reaction Kit (Applied Biosystems Inc, Foster City, CA, USA) according to the manufacturer's protocol. The DENV primers used for sequencing were designed based on the DENV-1 standard Hawaii strain (GenBank accession number: KM204119) and are presented in Table 1.

Ultimately, 17 strains causing CPE were observed, and 17 *E* gene sequences were obtained from the patient samples. We uploaded the sequences to GenBank and received their IDs: the accession numbers for the sequences are provided in Table 2.

## Data set

All the DENV-1 sequences (as of 2015) were downloaded from GenBank. The 3456 dengue sequences with known collection locations were retained from the 5483 sequences. The acquired and sample sequences were then aligned with ClustalX v. 2.0 software. CD-HIT v. 4.6 was used to reduce the redundancy, with a threshold level of 0.95. One sequence from each cluster was randomly chosen as a representative among sequences that shared a similar isolation location and time. A maximum likelihood (ML) tree consisting of 443 sequences was reconstructed under the generalized time-reversible (GTR) + gamma-distributed (G) + invariant sites (I) model in MEGA v. 6.0. We found 160 genotype I, 3 genotype II, 4 genotype III, 101 genotype IV and 175 genotype V sequences.

## Phylogenetic analyses of genotypes I and V

To identify the genotypes of the sample sequences, a ML tree was reconstructed with 120 sequences belonging to different genotypes and the sample ones: the sample sequences were found to belong to genotypes I and V. Another complete ML tree of genotype I and V was reconstructed with the GTR+G+I (genotypes I) and GTR+G (genotypes V) models, respectively, as described above.

Table 2. Newly isolated DENV-1 strains from Guangzhou during 2014. The GenBank accession numbers and the districts for the strains are shown

| Strain | Date of occurrence | GenBank accession number | District |
|---|---|---|---|
| GZ/E24 | 2014-10-31 | KT037099 | Yuexiu |
| GZ/E25 | 2014-11-10 | KT037100 | Yuexiu |
| GZ/E27 | 2014-11-20 | KT037101 | Lianshan |
| GZ/E31 | 2014-11-5 | KT037102 | Yuexiu |
| GZ/E101 | 2014-11-25 | KT037103 | Liwan |
| GZ/E99 | 2014-10-5 | KT037104 | Nanhai |
| GZ/E98 | 2014-10-5 | KT037105 | Yuexiu |
| GZ/E86 | 2014-10-23 | KT037106 | Panyu |
| GZ/E80 | 2014-10-29 | KT037107 | Yuexiu |
| GZ/E79 | 2014-11-3 | KT037108 | Baiyun |
| GZ/E66 | 2014-11-9 | KT037109 | Tianhe |
| GZ/E19 | 2014-11-20 | KT037110 | Baiyun |
| GZ/E6 | 2014-10-25 | KT037111 | Yuexiu |
| GZ/37 | 2014-10-31 | KT037112 | Yuexiu |
| GZ/E9 | 2014-10-27 | KT037113 | Yuexiu |
| GZ/E38 | 2014-11-3 | KT037114 | Yuexiu |
| GZ/E41 | 2014-11-9 | KT037115 | Tianhe |

### Phylogeography analyses of the local cases from 2013–2015

A total of 250 dengue sequences were downloaded from GenBank using the National Center for Biotechnology Information nucleotide BLAST program (as of 2016), which were compared with the study samples. The downloaded and sample sequences were then aligned with ClustalX v.2.0 software and 157 sequences were retained after removing duplicate nucleotide sequences. Bayesian evolutionary analysis of the local cases from 2013–2015 was performed in BEAST v. 1.8.2 software using the Bayesian Markov chain Monte Carlo (MCMC) method. The mean evolutionary rate of the *E* gene and the time to the most-recent common ancestor (tMRCA) were calculated through the best-fit model of nucleotide substitution that was selected by jModelTest and the relaxed molecular clock (uncorrelated lognormal). The MCMC sample was analyzed with 100 million replications and every sample was 10, 000 generations. The convergence of the data was assessed by TRACER v. 1.6 software with an effective sample size that was greater than 200. The maximum clade credibility (MCC) was conducted with burn-in of 10% by using TreeAnnotator v. 1.8.2 software. The MCC tree was visualized in FigTree v. 1.4.2 software.

## RESULTS

### Serotypes and genotypes of the predominant disease-causing DENV in Guangzhou in 2014

A total of 17 DENV serum samples from 168 showed pronouncedly cytopathic effects. A quick judgment on the serotypes was made with multiplex RT-PCR by determining the sizes of the amplicons via agarose gel electrophoresis. We verified that all 17 virus strains were DENV-1.

All of the sequence data for the different spatiotemporal DENV-1 sequences published were retrieved from GenBank to maximize the representation of the genotypes, regions and years of isolation. However, isolates that shared higher nucleotide sequence homology were winnowed out, so the final tree could be reconstructed with the most representative local samples for each genotype. The generalization of the ML method for the complete *E* gene sequences is shown in Figure 1.

Genotype I was extensively distributed in Southeast Asia (including Indonesia, Laos, Thailand, Myanmar, Malaysia, Singapore and Vietnam), the Middle East (including Saudi Arabia) and China. Genotypes II and III only circulated transiently in Thailand and Malaysia. Genotype IV mostly represented strains from the countries in the Pacific Rim (including Korea, China, Japan, Myanmar, Malaysia and Indonesia), The Western Pacific islands (for example, Nauru, French Polynesia, Hawaii and The Philippines) and Australia. Genotype V represented most DENV-1 strains collected from America, West Africa and Asia (Chen and Vasilakis, 2011). Genotypes I, IV and V, which were the main sources of the imported cases in Guangzhou, have been transmitted to Guangzhou more than once.

As illustrated in Figure 1, the 17 newly identified DENV-1 isolates were classified into genotypes I and V. Eight strains were clustered into genotype I and nine strains fell into genotype V; genotype I was also clustered into 2 branches. Two strains (KT037107 and KT037108) were intimately related to genotype I from Singapore in 2010 and Malaysia in 2009, while the six strains (KT037105, KT037106, KT037101, KT037103, KT037109 and KT037100) that were clustered together were closely related to the strains from Guangzhou in 2013 and Thailand in 2009. Nine genotype V strains (KT037112, KT037110, KT037113, KT037099, KT037102, KT037104, KT037111, KT037114 and KT037115) clustered together and shared high homology with strains from Singapore and Malaysia in 2013.

### Phylogenetic analyses of genotypes I and V

Using the phylogenetic tree, which is based on a large number of samples, we analyzed the transmission sources for genotype I and found that there were two sources. As shown in Figure 2, the 6 genotype I strains (KT037100, KT037101, KT037103, KT037105, KT037106 and KT037109) and the strains found in Guangdong in 2013, 2014 and 2015 clustered together. Therefore, we suspected that DENVs had formed an enzootic transmission cycle in Guangzhou. However, the Singapore strain from 2013 (KJ806945) was clustered in the same branch as above. Further research was planned to explore this. Another 2 genotype I strains (KT037107 and KT037108) were clustered with: strains from Guangzhou in 2004, 2005, 2007 and 2009; strains from Malaysia in 2005, 2008, 2012, 2013 and 2014; strains from Singapore in 2004, 2005, 2006, 2008, 2009 and 2014; strains from Indonesia in 2007, 2008, 2010, 2011, 2012 and 2013; as well as a strain from Taiwan in 2014. Therefore, we deduced that the ancestor viruses of these two strains were previously involved in epidemics in the Malaya Peninsula or Indonesia.

As demonstrated in Figure 3, 9 strains from genotype V (KT037099, KT037102, KT037104, KT037110, KT037111, KT037112, KT037113, KT037114 and KT037115) were clustered with: strains from Guangdong in 2009, 2010, 2011 and 2013; strains from Singapore from 2008, 2009, 2011, 2012 and 2014; a strain from Malaysia in 2013; strains from India in 2005 and 2008 to 2011; and a strain from the Kingdom of Bhutan in 2013. We estimated that the original strains for these 9 viruses were transmitted in Malaysia and India, while the strain formed in Guangzhou in 2014 was most possibly

Figure 1. DENV-1 phylogenetic tree. The phylogenetic tree was reconstructed by the ML method with a GTR+G+I model (general time-reversible model with gamma distribution and invariant sites) with MEGA 6.0 software. Bootstrap values were set for 1000 repetitions. The taxon names correspond to the GenBank accession number/location/isolation year. The background colors vary with each genotype. The red circles denote samples belonging to genotype I, and the red triangles denote samples belonging to genotype V. The reference strain sequences were derived from GenBank (www.ncbi.nlm.nih.gov).

introduced from Singapore; its affinis strains were introduced to Guangdong in 2009, 2010, 2011, 2013 and 2014.

## Phylogeography analyses of the local cases in 2013–2015

Using the MCC tree (Figure 4), we analyzed the Guangzhou strains from 2013 to 2015; the Singapore strain from 2013 (KJ806945) was clustered in the same branch, the strain's most likely ancestor position was Guangzhou. As a whole, these strains were clustered in the same branch with strains from Thailand in 2008, and their most likely ancestor position was Thailand and the tMRCA was 2006. Hence, we inferred that local strains derived from Thailand in 2006, then gradually developed an enzootic transmission cycle in Guangzhou, and had been transmitted to Singapore in 2013. The processes of phylogeographic diffusion are shown in Figure 5.

## DISCUSSION

In the past few years, the scale of dengue outbreaks in Guangzhou has become increasingly larger. In 2014, the number of patients and the frequency of outbreaks was at its peak for the past 20 years. Sang and colleagues analyzed the dengue epidemics in Guangzhou over the past 40 years and believe that they were caused by imported cases (Sang et al., 2015a). However, in our study, we found that the strains found in Guangdong, China, from 2013 to 2015 were clustered together. The MCC tree shows that the most likely ancestor position for these strains was Thailand and the tMRCA was 2006. Thus, we deduced that the viruses found in Guangdong might be from independent epidemics in Guangdong, China, which is a different conclusion from the one suggested by Sang and colleagues (Sang et al., 2015a). The formation of a viral enzootic transmission cycle in Guangzhou was closely connected with the wintering of mosquitoes carrying viruses. Firstly, as Guangzhou belongs to a subtropical zone, the often humid and hot climate with abundant precipitation increased the probability of wintering mosquitoes. Moreover, the high population density in Guangzhou largely increases the probability of the virus transmitting every other year. Hence, for sub-

Figure 2. DENV-1 genotype I phylogenetic tree. The phylogenetic tree was reconstructed with the ML method and a GTR+G+I model with MEGA 6.0 software. The cluster with the yellow background color is shown separately on the right. The blue background color indicates that the strains were isolated from China. The pink background color indicates that the strains were isolated from other countries. The samples are denoted by red dots.

sequent prevention and control of dengue, the enzootic transmission cycle needs to be carefully observed, as knowledge of the cycle will play an important role in generating strategies for preventing another epidemic. Furthermore, not only should imported viruses be prevented, but also the prevention of local viral outbreaks should be strengthened.

In our study, it was found that three kinds of serotype 1 strains co-prevailed. Furthermore, Zhao and colleagues found DENV serotype 2 strains in the dengue epidemic in Guangzhou in 2014 through isolation (Zhao et al., 2016). These two results prove that there were viruses of different serotypes and genotypes co-prevailing in the dengue outbreak in Guangzhou in 2014. Co-prevailing viruses lead to some people having mixed infections due to the presence of different DENV serotypes, which increase the patients' risk of DHF and DSS, and threaten patients' lives. Currently, serotype 1 is the main serotype in the Guangzhou dengue epidemic, where most of the cases are mild and secondary infections are infrequent. However, as the number of patients infected by DENV gradually increases, the risk of secondary infections will also increase.

Guangzhou has a humid subtropical climate that is influenced by the East Asian monsoon. Summers are wet with high temperatures, high humidity and a high heat index, which provides a perfect habitat for *Ae. albopictus* (DENV transmission vector). Sang *et al*. believed that Southeast Asia was the main source of the viruses spread in Guangzhou, which was once again proven in our study, as we found that some strains from genotype V and I epidemics in 2014 originated from Southeast Asia (2015). Why has Southeast Asia, specifically Guangzhou, become an area for imported dengue outbreaks? There are two main reasons. First, immigrants are drawn to Guangzhou, which is estimated to have a population of 13 million, and this is expected to increase in the foreseeable future. Second, for Guangzhou residents, some Southeast Asia countries, such as Thailand, Malaysia, Singapore and others, are popular tourist destinations.

Figure 3. DENV-1 genotype V phylogenetic tree. The phylogenetic tree was reconstructed with the ML method and a GTR+G model with MEGA 6.0 software. The cluster with the yellow background color is shown separately on the right. The blue background color indicates the strains isolated from China. The pink background color indicates that strains isolated from other countries. The samples are denoted by red triangles.

Thus, Southeast Asia continues to be the source of viruses that can promote dengue outbreaks in Guangzhou.

There are imported dengue cases in Guangzhou every year that do not trigger a large-scale endemic transmission every time, so why was there a dengue outbreak in 2014? We inferred that it was probably due to the climate that year, because climate significantly influences viruses, mosquitoes and hosts. A large number of studies on the effect of climate on dengue morbidity have been completed in China and abroad (Yang et al., 2016; Cheng et al., 2016; Ebi and Nealon, 2016; Sang et al., 2015; Quam et al., 2016). These studies indicated a preferred temperature range for DENV reproduction from 18.0 to 33.2 °C. In additional research, it was suggested that the number of dengue patients increases 6% for every 1 °C increase in temperature, and 61% for every 1 mm increase in rainfall (Ditsuwan et al., 2011). According to a number of studies, high temperatures and humidity probably lead to more frequent mosquito activities and benefit mosquito breeding (Yang et al., 2009; Sarfraz et al., 2014; Rohani et al., 2011). In addition, DENV-1 genotype I strains are seldom imported to Guangzhou, so antibodies were low in the people in Guangzhou, which probably benefited the DENV outbreak in Guangzhou. As the clinical spectrum of DENV is similar to those of flu, the number of DENV infectors we obtained was likely to be lower than the actual number, so the epidemic of DENV may have been much more serious than we observed. As a result, we should build an early warning system for dengue according to a comprehensive analysis of unusual climate conditions (like El Nino, etc.) and other factors.

Based on current molecular epidemiology information, dengue outbreaks in the world are affected by many factors, including economy, hygiene, climate and others. As a result, prevention and control should be emphasized according to the outbreak features in each region. Dengue outbreaks in Guangzhou are mainly caused by an enzootic transmission cycle and imported cases. Climate also has a major impact on dengue outbreaks in Guangzhou, which also display other characteristics, such as strong seasonality, high morbidity in old towns, high infection rates in adults and so on.

It is vital that a safe and effective vaccine is obtained for dengue prevention. In fact, some measures can be taken to prevent dengue. Anti-mosquito methods and cleaning-up mosquito eggs and breeding grounds have been efficient ways for preventing and controlling dengue in Guangzhou. Guangzhou is the Southern gateway of China in foreign trade and has extremely high population mobility. Based on geographic advantages, Guangzhou has especially close communication with

Figure 4. The local DENV-1 genotype I MCC tree. The MCC tree was visualized with FigTree v. 1.4.2 software. The blue background color indicates that strains were isolated from China. The green background color indicates that strains were imported from Thailand. The location in the clade indicates the most likely ancestor position. The time in the clade indicates the tMRCA.

Figure 5. The process of phylogeographic diffusion of the local cases detected in Guangzhou. (▬ ▬ ▬ ▬) The strains from Malaysia transmitted to Thailand and Laos in 2003; (··········) the strains from Thailand transmitted to Guangzhou and Singapore in 2006; (▬ · ▬ · ▬) the strains from Guangzhou transmitted to Singapore in 2013.

Southeast Asian countries, many of which are severely afflicted by dengue. Although precautions should be the main facets of dengue prevention and control, manpower and material resources need to be ready before dengue season to make sure that patients receive timely and effective treatments once cases occur.

DENVs of different serotypes and genotypes co-prevailed in the dengue outbreak in Guangzhou in 2014. Six genotype I strains were probably introduced to China from Thailand before 2009, widely transmitted in the epidemics in 2013 and 2014, and continued to circulate until 2015; one affinis strain was found in Singapore. This is indicative that in Guangzhou DENV had not only been imported, but also been gradually exported and had developed via an enzootic transmission cycle in Guangzhou. As for the other 2 genotype I strains, they were introduced to China from the Malaya Peninsula in 2014, while 9 genotype V strains were from Malaysia or India in 2014.

## ACKNOWLEDGMENTS

This work was supported by the Guangdong Natural Science Foundation (No. S2012030006598), the Science & Technology Planning Project of Guangdong Province of China (No. 2013A020229007), and the Innovative Program of the State Key Laboratory of Virology (No. 2016KF001). We would like to thank the China Center for Type Culture Collection for donating the C6/36 cells, and Jing Peng and Weiyong Liu from the Department of Clinical Laboratory, Tongji Hospital, Tongji Medical College, Huazhong University of Science and Technology, for their guidance on the phylogenetic analyses.

## AUTHOR CONTRIBUTIONS

JGW designed and supervised the experiments. GL and PP carried out the experiments. QYH, XJK, KLW and WZ analyzed the data. GL and PP wrote the paper. YTL, HTH, JBL and ZDZ performed sample collections. DW, XHL and XPL provided helpful suggestions about the study. All authors read and approved the final manuscript.

## COMPLIANCE WITH ETHICS GUIDELINES

The authors declare that they have no conflict of interest. This study was approved by the Guangdong Province Traditional Chinese Medical Hospital Ethical Review Committee (no. B2015-011-01). Informed oral consent was obtained from all of the patients recruited for the study.

## OPEN ACCESS

This article is distributed under the terms of the Creative Commons Attribution 4.0 International License (http://creative commons.org/licenses/by/4.0/), which permits unrestricted use, distribution, and reproduction in any medium, provided you give appropriate credit to the original author(s) and the source, provide a link to the Creative Commons license, and indicate if changes were made.
